# Effect of l-ornithine l-aspartate against thioacetamide-induced hepatic damage in rats

**DOI:** 10.4103/0253-7613.71926

**Published:** 2010-12

**Authors:** Abul K. Najmi, K.K. Pillai, S.N. Pal, M. Akhtar, M. Aqil, M. Sharma

**Affiliations:** Department of Pharmacology, India; 1 CH 1211, World Health Organization, Geneva 27, Switzerland, India; 2 Department of Pharmaceutics, Faculty of Pharmacy, Hamdard University, New Delhi 110 062, India

**Keywords:** Hepatopathy, ornithine aspartate, oxidative stress, thioacetamide

## Abstract

**Objective:**

To investigate the hepatoprotective activity of L-ornithine-L-aspartate against thioacetamide (TAA)-induced hepataopathy in rats.

**Materials and Methods:**

The hepatoprotective activity of L-ornithine-L-aspartate (OA) at a dose of 2 g/kg, p.o. for 10 days was evaluated against TAA (250 mg/kg, i.p. for 2 days) induced hepatopathy in rats. Biochemical parameters such as serum aspartate transaminase, alanine transaminase, alkaline phosphatase, bilirubin and glutathione, thiobarbituric acid reactive substances, and protein in liver tissues were estimated to assess the liver function.

**Results:**

TAA-induced pathogenic changes in the levels of the above indices were significantly (*P* < 0.01) reversed by the OA treatment. OA treatment also exhibited significant restoration of the hepatic architecture and lobular structure in histological evaluation of the rat liver sections.

**Conclusion:**

Ornithine aspartate exhibited significant hepatoprotective activity against TAA-induced hepatic damage in rats.

## Introduction

L-ornithine-L-aspartate (OA) is a stable salt of natural amino acids ornithine and aspartic acid. It has been studied extensively and proven to be effective in both experimental as well as clinical set up of hepatic encephalopathy (HE).[1,2] HE is caused by hyperammonemia[3,4] that may be caused by insufficient removal of ammonia from blood by liver due to its compromised functional status[5] or portacaval shunting.[6] Hyperammonemia leads to increased levels of ammonia in brain, which is responsible for the development of HE.[6,7] OA produces relief in the symptoms of HE by reducing elevated levels of ammonia both peripherally and centrally.[8,9] Although a number of studies are available for the action of OA on HE, data on its direct action on the liver are lacking.[10]

The present investigation was designed to evaluate the effects of OA on thioacetamide (TAA)-induced hepatopathy in rats. The rats with hepatic damage caused by TAA were chosen as an animal model for this study, because TAA is very frequently used to induce HE in rats.[11,12]

## Materials and Methods

### Animals

Adult Wistar strain albino rats (weighing, 150–200 g) were used for the study. Animals were supplied by Central Animal House Facility of Hamdard University and kept under standard laboratory conditions in polypropylene cages in 12-h light/dark cycle at 25 ± 2°C. Animals were provided with standard pellet diet (Lipton, India) and water *ad libitum*. All the procedures carried out on animals were approved by Institutional Animal Ethics Committee (JHAEC).

### Drugs and chemicals

OA was purchased from Systopic Laboratories, New Delhi, India. TAA and all other biochemicals and chemicals used were of analytical grade.

### Experimental protocol

Rats were randomly divided into three groups of six animals each. Groups II and III were administered two injections of TAA (250 mg/kg, i.p.) at 24 h interval on the first and second day. Group I served as normal control and received equivalent volume of vehicle. From the second day onward, Groups I and II received normal saline (1 mL/kg, p.o.) for 10 days. Group III was administered ornithine aspartate (OA, 2 g/kg, p.o.) for 10 days. On the twelfth day, blood was collected under light ether anesthesia and animals were killed by high dose of ether. Liver samples were also collected in ice-cold normal saline for histopathological and biochemical evaluation.

### Biochemical analysis

Serum alanine transaminase (ALT),[13] aspartate transaminase (AST),[13] alkaline phosphatase (ALKP),[14] bilirubin (BRN)[15] and liver tissue glutathione (GSH),[16] thiobarbituric acid reactive substances (TBARS),[17] and protein[18] were estimated according to the reported procedures.

### Histological studies

Histological liver sections were prepared by modified method of Luna *et al*.[19] as described previously.[20]

### Statistical analysis

The data presented as mean ± SEM were analyzed by one-way ANOVA followed by Dunnett’s post-test. The results were considered statistically significant if the *P*-value was 0.05 or less.

## Results

A significant (*P* < 0.01) increase in serum AST, ALT, ALKP, BRN, and tissue TBARS levels was observed in animals treated with TAA (Group II) as compared to normal control group (Group I). Levels of AST, ALT, ALKP, BRN, and tissue TBARS concentration increased by TAA treatment were decreased significantly (*P* < 0.01) by post-treatment of animals with OA (2 g/kg, p.o.) for 10 days. Moreover, the significant decrease (*P* < 0.01) in tissue GSH, which was observed in animals with TAA treatment (Group II) as compared to normal control (Group I), was significantly (*P* < 0.01) reversed by OA post-treatment. Blood GSH and liver protein concentrations were not altered significantly (*P* > 0.05) by any of the above treatments [Tables 1 and 2].

**Table 1:** Effects of ornithine aspartate post-treatment on serum parameters in thioacetamide-induced hepatic damage in rats

*Group*	*Treatment*	*ALT (IU/mL)*	*AST (IU/mL)*	*ALKP (IU/mL)*	*Bilirubin (mg%)*
I	NS	56.0 ± 2.463	111.33 ± 9.670	20.719 ± 2.185	0.473 ± 0.038
II	TAA + NS	158.5 ± 14.080**	257.0 ± 35.409**	220.203 ± 6.425**	1.233 ± 0.142**
III	TAA + OA	84.0 ± 17.390**	123.90 ± 9.757*	36.215 ± 1.908**	0.590 ± 0.086**
F-ratio		11.811	8.340	62.86	13.187

Data expressed as mean ± SEM, *n* = 6; Significance of difference was evaluated with respect to Group II by one-way ANOVA followed by Dunnett’s *post hoc* test

(*P < 0.05,

** *P* < 0.01, ns = nonsignificant); NS = Normal saline; TAA = thioacetamide; OA = ornithine aspartate; ALT = Alanine transaminase; AST = aspartate transaminase; ALKP = alkaline phosphatase.

**Table 2:** Effects of ornithine aspartate post-treatment on liver tissue biochemicals in thioacetamide-induced hepatic encephalopathy in rats

*Group*	*Treatment*	*Blood GSH (mg%)*	*Tissue GSH (μmol/g of liver)*	*Tissue TBARS (nmol MDA/mg protein)*	*Protein (mg/g wet tissue)*
I	NS	1.197 ± 0.130	29.141 ± 1.228	0.126 ± 0.023	19.66 ± 1.253
II	TAA + NS	1.017 ± 0.133^ns^	9.841 ± 1.561**	0.676 ± 0.048**	16.26 ± 1.305^ns^
III	TAA + OA	1.306 ± 0.130^ns^	20.522 ± 2.191**	0.189 ± 0.026**	15.2 ± 0.935^ns^
F-ratio		0.868	25.247	57.665	4.390

Data expressed as mean ± SEM, *n* = 6; Significance of difference was evaluated with respect to Group II by one-way ANOVA followed by Dunnett’s *post hoc* test

(*P < 0.05,

** *P* < 0.01,

ns = non significant); NS, normal saline; TAA, thioacetamide; OA, ornithine aspartate; GSH, glutathione; TBARS, thiobarbituric acid reactive substances; MDA, malondialdehyde.

### Histopathological observations

**Figure 1:**
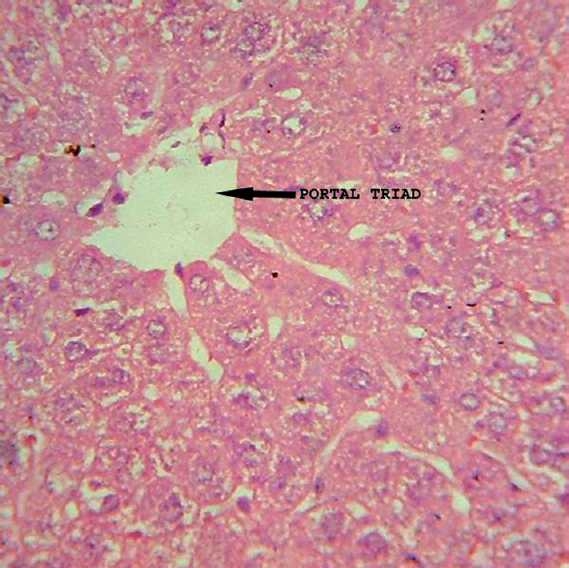
Group I: Liver section of normal control rats showing normal hepatic architecture and lobular structure (H and E, ×400).

**Figure 2:**
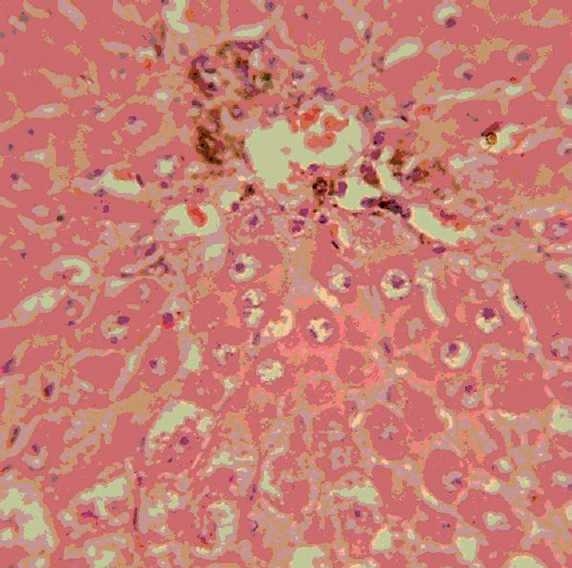
Group II: Liver section of thioacetamide-treated rats, showing periportal hepatocytic vacoulation, clearing of cytoplasm, pigmentation and scattered inflammation, necrosis, and giant cell transformation (H and E, ×400).

**Figure 3:**
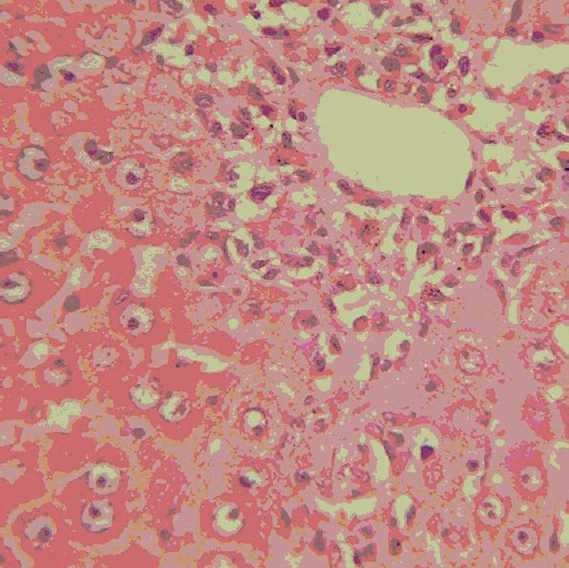
Group III: Liver section of rats treated with thioacetamide + ornithine aspartate showing only mild inflammation and pigmentation, but no necrosis (H and E, ×400).

Histology of the liver sections of normal control animals (Group I) showed normal hepatic architecture and normal liver lobular structure with well-preserved cytoplasm, prominent nucleus, and nucleolus [Figure 1]. The liver sections of TAA-treated animals (Group II) showed hepatic cells with severe toxicity characterized by centrilobular necrosis, periportal hepatocytic vacoulation with clearing of cytoplasm, heavy pigmentation around central veins, scattered inflammation, and giant cell transformation [Figure 2]. OA post-treatment (Group III) appeared to significantly reduce the TAA-induced toxicity as evidenced by less inflammatory changes, less pigmentation, and no necrosis [Figure 3].

## Discussion

TAA was originally used as a fungicide to protect oranges from decay. In the aftermath of its inadvertent use, it was soon recognized as a potent hepatotoxin and carcinogen in rats.[21] The compound has also been reported toxic to kidney and thymus.[22] It is also reported to induce symptoms comparable to experimental partial hepatectomy. Cyt-P450 system is known to metabolize TAA in rat liver. Mechanism of toxicity is postulated to be due the formation of thioacetamide-*S*-oxide which causes change in cell permeability, increases in intracellular Ca^++^ concentration, increases in nuclear volume and enlargement of nucleoli, and inhibition of mitochondrial activity which leads to cell death.[23] TAA also increases the BRN levels in serum,[24] but decreases the protein contents in the liver by inhibiting incorporation of amino acids into liver protein.[25]

In a number of animal models, TAA-induced cirrhosis seems to resemble the important features of human disease.[26] Elevated levels of serum enzymes are indicative of cellular leakage and loss of functional integrity of the cell membrane in liver.[27] Damage to liver cells cause leakage of cellular enzymes into serum. Significant rise in levels of AST, ALT, and ALKP could be taken as an index of liver damage. TAA is known to cause changes in cell permeability by its metabolite thioacetamide-*S*-oxide.[23] In our study, the rise in AST, ALT, and ALKP levels induced by TAA administration was significantly reduced by OA post-treatment, suggesting that its protective activity might be due its effect against cellular leakage and loss of functional integrity of the cell membrane in hepatocytes. TAA is also reported to elevate BRN concentration[24] by damaging hepatic cells. In this study also, TAA treatment increased serum BRN concentration significantly, which was reversed significantly in rats treated with OA.

GSH is an important endogenous antioxidant system that is found in particularly high concentration in liver, and it is known to have key functions in protective processes. The reduced form of GSH becomes readily oxidized to glutathione disulfide (GSSG) on interacting with free radicals. Excessive production of free radicals resulted in the oxidative stress, which leads to damage of macromolecules, e.g. lipids and can induce lipid peroxidation *in vivo*.[28] In our study, TAA depleted tissue GSH and increased tissue TBARS significantly. The post-treatment of animals with OA significantly reversed the TAA-induced changes of these oxidative stress markers.

Histologically, TAA toxicity leads to the prominent changes in the liver tissue architecture including the appearance of necrotic cells and inflammatory cells, mostly macrophages around the central vein. TAA produces centrilobular necrosis with marked accumulation of lipids on acute exposure.[23] In our study, TAA administration produced severe periportal inflammation with pigment deposition and also scattered inflammatory cell infiltration in the hepatic lobule. The post-treatment of animals with OA, reversed significantly TAA-induced pathogenic changes in liver. A mild periportal inflammation with no pigmentation or necrosis was observed in the liver sections of animals treated with TAA and OA.

OA therapy is one of the key treatments of the HE. A number of mechanisms have been attributed to its action in HE. It has been proven to lower blood and brain ammonia concentration in both clinical and experimental set up.[1,8,9] Hyperammonemia is considered to be a major player in the pathogenesis of HE.[4] The infusion of OA has also been shown to reduce the plasma levels of aromatic amino acids such as phenyalanine, tyrosine, etc., in a dose-dependent manner.[29] Increased aromatic amino acid concentration in CNS has been implicated in the synthesis of false neurotransmitters such as phenyl ethanolamine, octopamine, etc. (instead of dopamine, norepinephrine, etc.), which leads to disturbance in normal physiological neurotransmission during HE.[30]

This study suggests that OA-induced alleviation in the symptoms of HE may be due to the overall improvement in the efficiency of liver functions. Most of the hepatic damage caused by TAA in rats is due to the oxidative stress it produces in the liver. Our results also suggest that OA treatment could control the TAA-induced oxidative stress by (i) strengthening the endogenous antioxidant defense and reducing thiobarbituric acid reactive species and (ii) stabilizing hepatic cell membrane leading to maintenance of cell membrane integrity, which further assists in reducing oxidative stress.
